# Coping with the cold and fighting the heat: thermal homeostasis of a superorganism, the honeybee colony

**DOI:** 10.1007/s00359-021-01464-8

**Published:** 2021-02-17

**Authors:** Anton Stabentheiner, Helmut Kovac, Monika Mandl, Helmut Käfer

**Affiliations:** Institute of Biology, University of Graz, 8010 Graz, Austria

**Keywords:** Honeybee colony, Thermoregulation, Temperature, Behaviour, Thermography

## Abstract

The worldwide distribution of honeybees and their fast propagation to new areas rests on their ability to keep up optimal ‘tropical conditions’ in their brood nest both in the cold and in the heat. Honeybee colonies behave like ‘superorganisms’ where individuals work together to promote reproduction of the colony. Social cooperation has developed strongly in thermal homeostasis, which guarantees a fast and constant development of the brood. We here report on the cooperation of individuals in reaction to environmental variation to achieve thermal constancy of 34–36 °C. The measurement of body temperature together with bee density and in-hive microclimate showed that behaviours for hive heating or cooling are strongly interlaced and differ in their start values. When environmental temperature changes, heat production is adjusted both by regulation of bee density due to migration activity and by the degree of endothermy. Overheating of the brood is prevented by cooling with water droplets and increased fanning, which start already at moderate temperatures where heat production and bee density are still at an increased level. This interlaced change and onset of different thermoregulatory behaviours guarantees a graded adaptation of individual behaviour to stabilise the temperature of the brood.

**Supplementary Information:**

The online version contains supplementary material available at 10.1007/s00359-021-01464-8.

## Introduction

The honeybee (*Apis mellifera*) displays advanced regulation of the nest climate, in summer as well as in winter (Hess 1926; Himmer 1932; Simpson 1961; Kronenberg and Heller 1982; Southwick 1983, 1985; Seeley 1995; Stabentheiner et al. 2003a). Thermal homeostasis of the colony is especially important for the brood (Koeniger 1978; Bujok et al. 2002; Kleinhenz et al. 2003; Stabentheiner et al. 2010), because honeybee larvae and pupae are extremely stenothermic (Tautz et al. 2003; Groh et al. 2004; Petz et al. 2004; Jones et al. 2005; Becher et al. 2009). A brood nest temperature of 32–36 °C guarantees a high and constant development speed (Petz et al. 2004). Accordingly, the accuracy of thermoregulation is high in the presence of brood, and much more variable and generally lower in colonies without brood (Koeniger 1978; Ritter 1982; Southwick 1985; Bujok et al. 2002; Kleinhenz et al. 2003; Stabentheiner et al. 2010). While eggs and larvae (in open brood cells) can tolerate also somewhat lower temperatures for some time, the pupae (in sealed brood cells) are very sensitive to cooling. If pupae are exposed to temperatures lower than 32 °C for too long there is a high incidence of malformations of wings, legs and abdomen (Himmer 1932). Adults may also suffer from behavioural and neural insufficiencies especially if temperatures are too low (Tautz et al. 2003; Groh et al. 2004; Jones et al. 2005; Becher et al. 2009) but also if they are too high during development (Medina et al. 2018; McAfee et al. 2020). Since the brood lacks regulatory ability and does not produce enough heat by itself for proper development (Melampy and Willis 1939; Allen 1959; Petz et al. 2004), achievement of thermal constancy in a variable environment has to be accomplished by the worker bees (Koeniger 1978; Bujok et al. 2002; Kleinhenz et al. 2003; Stabentheiner et al. 2010). Warming behaviour is triggered by chemical and tactile stimuli of the brood, and sealed cells were reported to be more attractive than open ones (Koeniger 1978).

In research on the thermoregulation of breeding honeybee colonies, one has to look at them as a ‘superorganism’ (Southwick 1982, 1983; Moritz and Southwick 1992; Heinrich 1993) where thousands of individuals work together to achieve optimal conditions for the development of the brood. Investigations on the contribution of individuals to colony heat production, in reaction to variation in environmental temperature, had to be performed in observation hives with usually only two combs and far fewer bees than in a standard colony. While observation hives are well suited to investigate the mechanisms of temperature homeostasis (Bujok et al. 2002; Kleinhenz et al. 2003; Stabentheiner et al. 2010), they have a considerably higher heat loss than standard commercial bee hives or naturally nesting colonies (Mitchell 2016, 2019). Therefore, an unnaturally high incidence of endothermic bees, which produce heat with their flight muscles, has to be expected to stabilise the in-hive microclimate (Stabentheiner et al. 2010). Only in a colony of standard size and type, the rate and degree of endothermy can be expected to be equivalent to natural conditions. Such measurements have been carried out in winter clusters (Stabentheiner et al. 2003a) but are still missing in breeding summer colonies. According to common rules of biological and technical cybernetics, we hypothesised that a breeding colony will have to establish regulatory stability by counteracting heating and cooling mechanisms.

Thermal stress for a honeybee colony does not only occur in a cold ambience but also at high temperatures (Lindauer 1954; Abou-Shaara et al. 2017). In scenarios of future global warming, increased environmental temperatures are expected to challenge also honeybee colonies (Kovac et al. 2014; Bordier et al. 2017; Medina et al. 2018; Kablau et al. 2020; McAfee et al. 2020). If the hive is in danger of being overheated the bees cool it by fanning (Southwick and Moritz 1987; Sudarsan et al. 2012; Cook and Breed 2013; Egley and Breed 2013; Cook et al. 2016), and they collect water to spread it on the combs (Lindauer 1954; Kühnholz and Seeley 1997). The supply with water is the task of water gatherers (Lindauer 1954; Kühnholz and Seeley 1997; Visscher et al. 1996; Schmaranzer 2000; Kovac et al. 2010, 2018). We here not only demonstrate the onset of this behaviour in dependence on heat stress but also show the spatial distribution of cooling droplets.

If one looks at a colony as a ‘homeothermic’ superorganism (Moritz and Southwick 1992) a main question is, whether it is possible to define critical temperatures (*T*_c_) limiting a thermoneutral zone, below or above which the individuals show increased metabolic efforts to stabilise the core temperature, comparable to homeotherms like mammals and birds (Morgan 1998; Willmer et al. 2000). Unlike in a multicellular organism, however, the individuals of the superorganism honeybee colony have more degrees of freedom of how to react to temperature changes than body cells. They can react by behavioural or metabolic adaptation. If it is to achieve a deeper understanding of their contribution to colony homeostasis, quantification of their individual metabolic effort is necessary. Unfortunately, inside a colony direct measurement of metabolic activity of individuals is not possible without disturbing their behaviour, which can increase metabolic activity by a factor of up to 100 (Crailsheim et al. 1999; Stabentheiner and Crailsheim 1999). Measurement of the body temperature of bees by infrared thermography, therefore, was the method of choice to assess endothermic activity in undisturbed honeybees (Stabentheiner et al. 2003a, b). Our investigation of the thermal relationships and bee density allows quantification of regulatory mechanisms in a colony of standard size and shape in reaction to environmental changes.

## Materials and methods

### Colonies, bee treatment and experimental procedure

A queenright colony of *Apis mellifera carnica* Pollmann (approximately 8000–9000 bees) was established in spring on 10 wax foundations, placed in a (acrylic)glass hive (5 mm wall thickness) insulated by polystyrene (20 mm), which was set up in an air-conditioned laboratory. The bees had free access to the outside (Fig. S1a). Bees got about 2 months to build the combs and start breeding. During this time, they were provided with sugar solution (50%). To achieve the desired environmental temperatures (*T*_e_), we heated or cooled the hive and the laboratory by an air stream directed to the top of the experimental hive (Fig. S1a). Below the hive, the air was withdrawn to have a stable as possible regulation of the environmental temperature (*T*_e_ ~  ± 2 °C). Measurements started when capped brood covered most of the central combs (Fig. S1b). The hive could be opened between the two central combs on low-friction hinges. As opening of the hive would have disturbed the bees, two plastic films transparent to infrared radiation covered the two central combs. This way, the bees remained very quiet when we opened the hive because they were not separated from each other, and cooling of the cluster interior was minimised during thermographic measurements. Four small windows (each 2 cm × 3 cm) in the edges of the films allowed the bees to communicate and to move between the two combs (Fig. S1d).

The bees could leave and enter the hive through a 1-m-long plastic tube leading outside, with 5 cm inner diameter. We determined the air temperature near the bees (*T*_a_) by triangular interpolation from a mesh of 24 NiCr/Ni-thermocouples at a height of 5–9 mm above one central comb. A temperature/humidity (T/rH) sensor was mounted in the empty space 8 cm below the lower frame edge (AHLBORN FHA646-R). Additional 11 thermocouples measured the air temperature in the centre between the other combs and above the outer comb surfaces (Fig. S1c). The actual bee position on the central comb during measurement was determined relative to a wire mesh with 3 × 3 cm rectangles mounted at a height of 10 mm above the comb. By dividing each mesh rectangle into 5 subsections (edges: a–d, and centre: e; Fig. S1d), we determined the position of the bees with a resolution of ± 10 mm. Temperature and humidity data, and the readout of an infrared reference radiator were stored every 5 s on a laptop computer via a data logger network (ALMEMO 5590-2, 40 channels; ALMEMO 2290-8, 5 channels; Ahlborn, Holzkirchen, Germany).

To visualise the bees’ thermoregulatory reaction under even more extreme cold stress, thermograms of a standard two-frame observation hive with about 3000 bees were taken two hours after opening it, with the combs just covered by an infrared-transmitting film.

### Body and comb temperature measurement

To have comparable hive conditions concerning brood size and number of workers, we conducted the measurements within a period of 7 days (11 to 18 July 2002). Thermographic measurements were carried out at daytime between 10:00 and 19:30 by quickly but gently opening the beehive between the central combs. After 30 s of infrared temperature recording, the hive was closed. Between the measurements the colony got at least 90 min or a whole night to calm down. After the measurements, once per day, we documented the exact ranges of brood cells, honey and pollen stores, and empty cells on a cell map. To cover the environmental temperatures (*T*_e_) relevant for honeybee colonies in temperate zone summer seasons, thermal colony homeostasis was investigated at 6 temperature ranges, average values amounting to 13.9, 18.8, 24.3, 28.3, 32.2 and 40.6 °C. At each environmental temperature, three measurements were made, except at the lowest one where we made 5 measurements.

Thermography allowed measurement of the body surface temperature without impairment of behaviour. Surface temperatures of the dorsal body of *all* bees on the central comb, of the comb beside each bee, and of water droplets (if present) were measured within a few seconds after opening the hive with a ThermaCam SC2000 NTS infrared (IR) camera with a standard lens (Fig. S1a; FLIR, Inc.; 320 × 240 pixel sensor, thermal resolution < 0.1 °C). During most of the experiments, bees were also hanging in a cluster below the comb (compare Fig. S1b, Fig. 2). Selected surface bees of this cluster sitting in an appropriate position were measured 5–15 s later by moving the IR-camera down on the supporting tripod. The IR-camera was calibrated for offset errors against a self-constructed miniature Peltier-element driven reference source of known temperature and emissivity (Fig. S1b) (Stabentheiner et al. 2012). Attenuation of the IR radiation by the plastic film covering the central comb was compensated for by changing the atmospheric transmission value during evaluation. Using an infrared emissivity of 0.97 of the honeybee cuticle (Stabentheiner and Schmaranzer 1987) and of 0.95 of the comb wax, surface temperature was measured to the nearest 0.7 °C. Thermographic data were stored digitally with 14-bit resolution on a portable computer at a rate of 50 Hz (DOLCH FlexPac-400-XG). This facilitated recognition of bees by their movement, which was especially important at the higher experimental temperatures where temperature differences were small and bees not easily identifiable. The presence of water droplets seen as dark (cool) spots in the thermograms was verified exemplary by visual inspection with a goose-neck lamp after the measurements. Thermograms of the standard two-frame observation hive were taken on a FLIR T650sc camera (640 × 480 pixel sensor, thermal resolution < 0.02 °C).

### Data evaluation

Evaluation of the surface temperatures of head (*T*_head_), thorax (*T*_thorax_) and abdomen (*T*_abdomen_) and of the comb beside the bees (*T*_comb_) was done from the stored files after the measurements, with AGEMA Research software (FLIR) controlled by a custom programmed Excel (Microsoft Corporation) VBA macro. This macro extracted temperature data necessary for exact temperature calculation at the thermographic measurement points from the logger files and interpolated them over time. With another Excel macro, we calculated the local air temperature at the actual positions of the bees (*T*_a_) by triangular interpolation between adjacent of the 24 thermocouples on the central comb and the T/rH-sensor in the space below it (Fig. S1d). Isotherm functions were calculated by Renka–Cline interpolation of thermocouple or comb surface temperatures with ORIGIN (OriginLab Corporation). To estimate isotherms, this gridding method generates a matrix from randomly distributed XY-data, by (a) triangulation of nearby XY-data points (as equiangular as possible), (b) estimation of gradients in *X* and *Y* direction for every nodal point as partial derivation of a quadratic function, and (c) calculation of an interpolated value for every point P by the use of the data points and the estimated gradients on each of the edges of the triangle containing a point P.

To separate endothermic from ectothermic bees, we classified them according to the thermal relationships of body parts (Stabentheiner et al. 2003a). Based on camera sensitivity (0.1 °C), body parts were considered as different if their temperatures differed by at least 0.2 °C. Though this partly interferes with temperature gradients on the comb, it is appropriate if it comes to simultaneously judge endothermy without behavioural disturbance in a large number of bees. Bees were considered as surely endothermic if the thorax was the warmest body part (*T*_hd_ < *T*_th_ > *T*_ab_). The class with *T*_hd_ = *T*_th_ > *T*_ab_ was counted as ectothermic though very weak endothermy in part of the bees could not be excluded. In all other classes, true ectothermy was assumed.

Heat production of the bees on the central comb was estimated on the basis of the resting metabolism of ectothermic bees (compiled from Kovac et al. 2007, 2014), and by the use of simultaneous measurements of energy turnover (*E*) and body temperature of endothermic foragers, according to the relation *E*/(*T*_body_ − *T*_a_) = -1.56347 + 0.38581 × *T*_a_ (mW/°C) (compiled from Stabentheiner and Kovac 2014), multiplied by the difference of mean *T*_body_ − T_a_ and the number of bees.

Statistics was done with the Statgraphics package (Statistical Graphics Corporation) or with self-written Excel sheets according to Sachs (1997). Correlations were calculated with Statgraphics or ORIGIN. Piecewise two- or three-segment linear regression was done with ORIGIN to define setpoints (start values) of behaviours or thermal relations on a statistical basis if standard linear or nonlinear regressions did not allow this adequately. Two-segment linear regression: *y* = *a*1 + *k*1 × *x* {for *x* < *xi*}; *y* = *yi* + *k*2 × (*x − xi*) {for *x* ≥ *xi*}, *yi* = *a*1 + *k*1 × *xi*; *xi*: intersection point of regressions. Three-segment linear regression: *yi*1 = *a*1 + *k*1 × *xi*1; *yi*2 = *yi*1 + *k*2 × (*xi*2 − *xi*1); if (*x* < *xi*1) *y* = *a*1 + *k*1 × *x*, else if (*x* < *xi*2) *y* = *yi*1 + *k*2 ×  (*x − xi*1), else *y* = *yi*2 + *k*3 × (*x* − *xi*2). *xi*1, *xi*2: intersection points of regressions. For *χ*^2^ statistics, the significance level was adjusted according to the Bonferroni correction for multiple comparisons wherever applicable (Sachs 1997).

## Results

### General thermal relationships

**Fig. 1:**
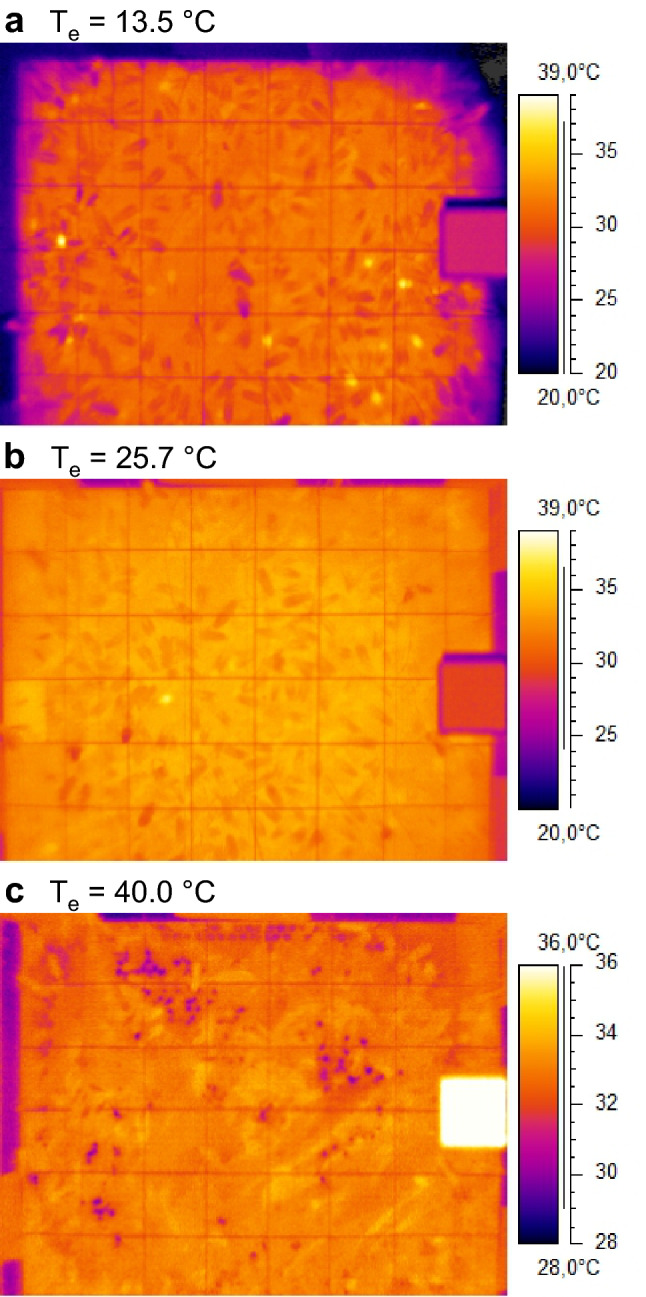
Sample thermograms of the central comb of a standard colony at various environmental temperatures (*T*_e_). Note intensely endothermic bees (yellow spots) in **a**, and dark spots in **c** where bees had spread water for cooling of the comb. Lines: wire mesh for position determination; right-hand squares: reference radiator for camera calibration. For more thermograms see Fig. S2

Typical thermograms of the investigated central comb containing mainly capped brood cells showed some intensely endothermic bees at low environmental temperatures of ~ 13–15 °C and ~ 18–19 °C (light yellow and white spots in Fig. 1a, Fig. S2a,b) but considerably fewer than in poorly insulated observation hives (compare Stabentheiner et al. 2010). At higher temperatures, endothermic bees were only identifiable by detailed evaluation of the thermograms.

**Fig. 2:**
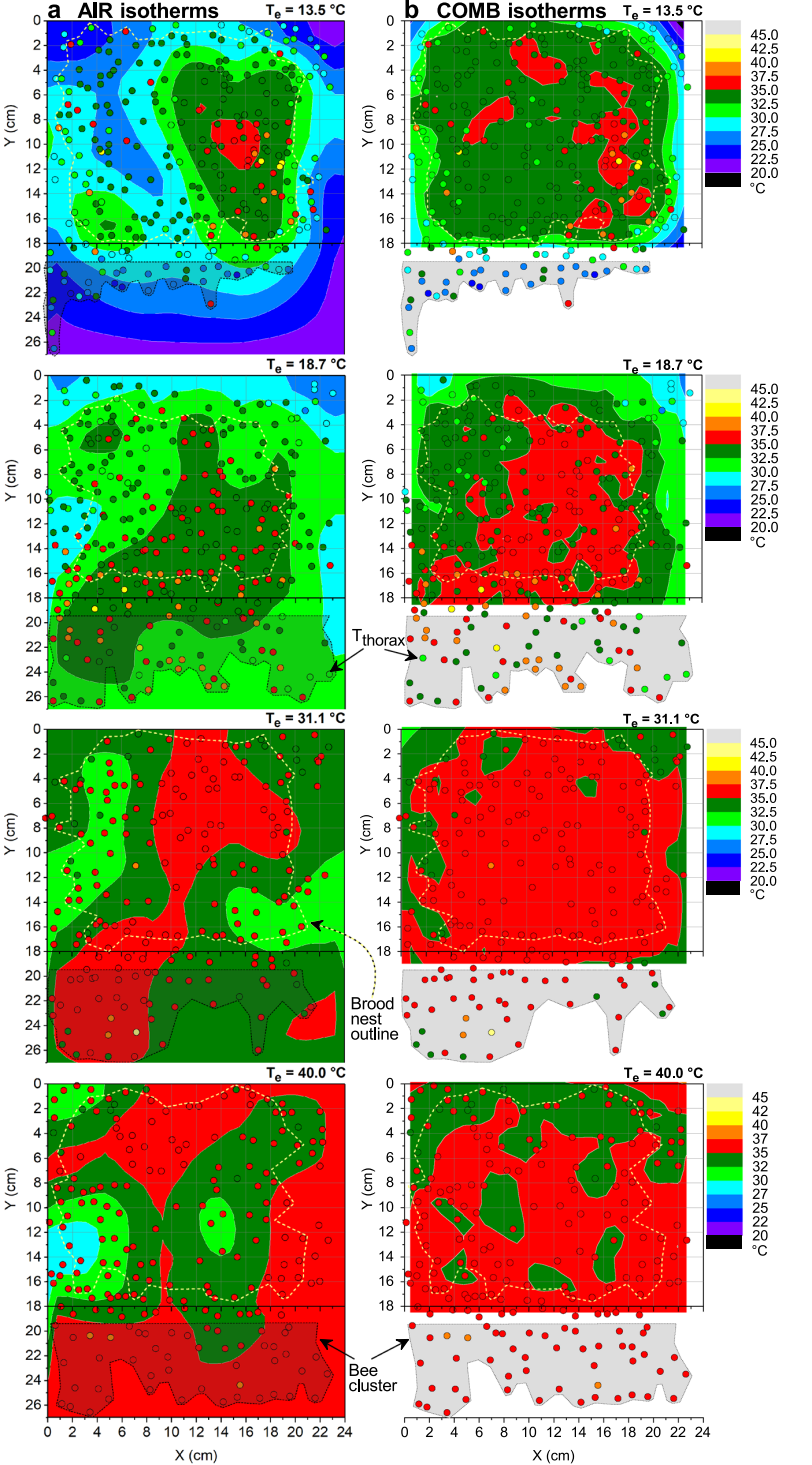
Distribution of thorax surface temperature (dots) in relation to **a** air temperature, and **b** surface temperature of the central comb, at selected environmental temperatures (*T*_e_). Isotherms bordering ranges of similar temperatures were calculated by Renka–Cline interpolation of thermocouple data (**a**) or of thermographically measured comb surface temperatures (**b**). In the bee clusters (grey areas) below the comb representative surface bees were measured. Broken yellow lines show brood nest dimension. White spots at *T*_e_ = 40 °C indicate main areas of depressed comb temperature due to the spreading of water droplets (see Fig. 1, Fig. S2)

**Fig. 3:**
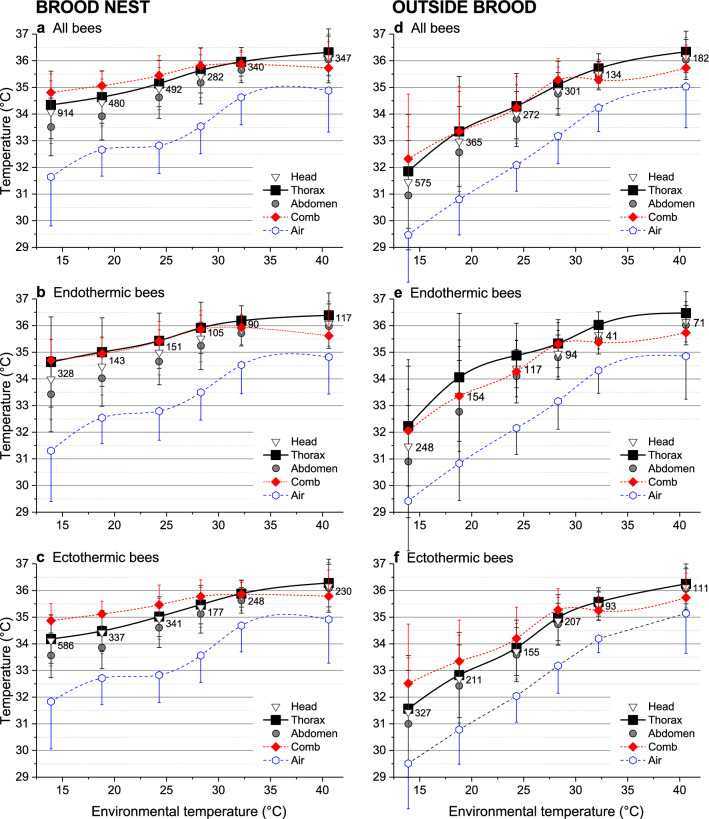
Body surface temperature of all bees present on the central comb, and of the comb and air temperature beside them in dependence on environmental temperature *T*_e_ (means of all measurements, with SD and *N*). Bees classified as endothermic or ectothermic according to Fig. 4. *T*_thorax_ different from *T*_comb_ and *T*_air_ at *P* < 0.0001, except (n.s.) *T*_thorax_:*T*_comb_ at *T*_e_ = 13.9–32.2 °C in (**b**), at *T*_e_ = 32.2 °C in (**c**), at *T*_e_ = 24.3–28.9 °C in (**d**), and at *T*_e_ = 13.9 °C and 28.3 °C in (**e**). For detailed statistics see Table S2

A main goal of our investigation was to relate the body temperature measurements to the local air and comb temperature. Figure 2 shows samples of the spatial distribution of both the air temperature (*T*_a_) and the comb surface temperature (*T*_comb_) across the central comb during such measurements. At the lowest environmental temperature (*T*_e_ = 13.5 °C), a steep gradient of the air temperature was observed across the brood nest. Only in its centre, the bees kept *T*_a_ above 35 °C (Fig. 2a). Comb temperature, by contrast, was more uniform and kept higher than 32.5 °C throughout the brood nest (Fig. 2b). At higher environmental temperatures the distribution of in-hive air and comb temperatures became more uniform, comb temperatures higher than 35 °C reaching its largest extension in the sample calculated at *T*_e_ = 31.1 °C. Both *T*_a_ and *T*_comb_ were still highest in the brood nest centre. However, during high heat stress at *T*_e_ = 40 °C, the relationships changed. The air temperature was now higher in a large part of the comb periphery (Fig. 2a, bottom graph). Despite the high peripheral air temperature, however, the comb temperature was kept below 35 °C in a considerable part of the comb (Fig. 2b, bottom graph). The bees accomplished this mainly by spreading of water droplets across the comb (see Figs. 1c, 2b bottom graph; Fig. S2e,f; for details see below). The thermal conditions in the colony, however, were not static. Supplementary Video S1 demonstrates the fluctuations of air temperature on the central comb within a period of 95 min. Body temperatures were on average higher than the local air temperature in the bee spaces (*T*_a_) (Figs. 2, 3).

A basic question in honeybee thermoregulation is the actual accuracy of colony homeostasis if ambient conditions change. Most accurately regulated was the temperature of the capped brood cells (mean *T*_comb_ = 34.8–35.9 °C), being lowest at a mean *T*_e_ of 13.9 °C and highest at 32.2 °C (Fig. 3a). At *T*_e_ = 40.6 °C, mean *T*_comb_ amounted to 35.7 °C. The air temperature in the bee space was always by about -3 to -0.7 °C lower than the comb temperature (from lowest to highest *T*_e_). Mean honeybee thorax temperatures (*T*_thorax_) ranged from 34.3 to 36.3 °C on the brood nest, being lower than the comb temperature below *T*_e_ = 32 °C but higher at *T*_e_ = 40.6 °C (Fig. 3a). Temperatures of head and abdomen were always lower on average. Outside the brood nest, temperature gradients were generally higher, and temperatures changed considerably stronger with *T*_e_ (Fig. 3d). Mean comb temperature increased from 32.3 to 35.7 °C, *T*_thorax_ increased from 31.9 to 36.3 °C, and *T*_a_ increased from 29.5 to 35.0 °C from lowest to highest *T*_e_, respectively. For statistical differences see Table S2.

### Endothermy and ectothermy

**Fig. 4:**
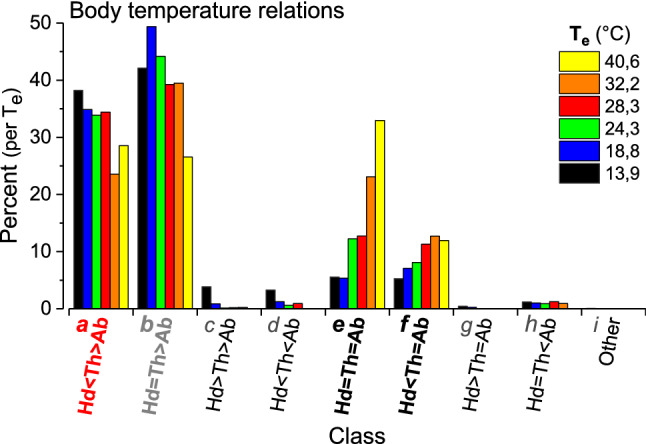
Percentage of bees classified as surely endothermic (class ***a***: *T*_head_ < *T*_thorax_ > *T*_abdomen_; minimum difference 0.2 °C) and as ectothermic (classes ***b–i***) at the different environmental temperatures (*T*_e_). In class ***b*** weak endothermy in part of bees could not be excluded. Distributions (*a–i*) different between *T*_e_ classes at *P* < 0.002 (*χ*^2^ = 77.45, df = 40, Bonferroni–Holmes correction: *τ* = 15). Classes *a* and *b* different from class *e* at *P* < 0.001 (*χ*^2^ = 34.78 and 41.74, df = 5)

**Fig. 5:**
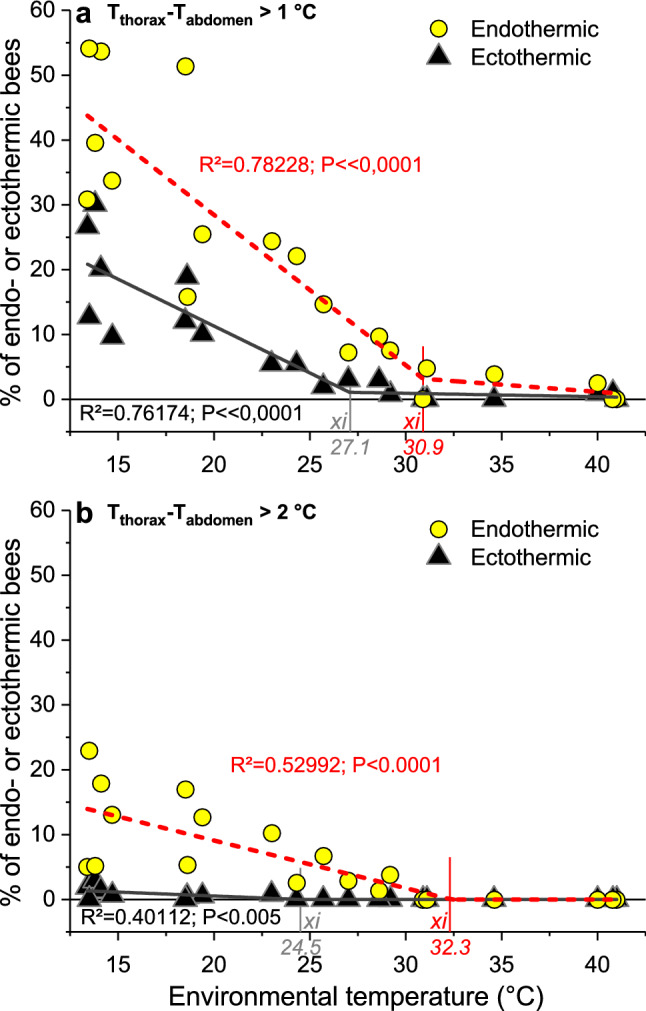
Percentage of bees classified as endothermic (class *a* in Fig. 4) or classified as ectothermic (classes *b*–*i* in Fig. 4) with a temperature difference between thorax and abdomen of more than 1 °C (**a**) or 2 °C (**b**), at different environmental temperatures (*T*_e_). Interpolation with piecewise two-segment linear function: *y* = *a*1 + *k*1 × *x* {for* x* < *xi*}; *y* = *yi* + *k*2 × (*x − xi*) {for* x* ≥ *xi*}, *yi* = *a*1 + *k*1 ×* xi*; *xi*: intersection point of regressions. Parameters: **a** *endothermic*: *a*1 = 74.81297, *k*1 = -2.31824, *xi* = 30.9, *k*2 = -0.22253; *ectothermic*: *a*1 = 40.22261, *k*1 = -1.44592, *xi* = 27.06495, *k*2 = -0.05247; **b** *endothermic*: *a*1 = 23.81831, *k*1 = -0.73642, *xi* = 32.34319, *k*2 = 1.29814×10^–10^; *ectothermic*: *a*1 = 2.92988, *k*1 = -0.11976, *xi* = 24.46544, *k*2 = 1.00438×10^–15^. *N* = 20 measurements, *R*^2^ = adj. for df

Another important question was of how many bees are engaged in active, endothermic heat production. To separate the endothermic from the ectothermic bees, we classified them according to the relation of body part temperatures (Fig. 4) (Stabentheiner et al. 2003a). Bees were classified as endothermic in case of *T*_head_ < *T*_thorax_ > *T*_abdomen_, with a minimum difference of 0.2 °C (class *a* in Fig. 4). The thorax temperature of bees classified as endothermic was on average higher than in bees classified as ectothermic both on the brood nest (Fig. 3b, c) and outside it (Fig. 3e, f), except at the highest *T*_e_. The relative number of endothermic bees (class *a* in Fig. 4) did not decrease much up to a *T*_e_ of 28.3 °C (38–34%). Only at higher temperatures a slight reduction to 23.5–28.5% was observed (class *a* in Fig. 4), similar to the bees which were classified as probably ectothermic (or only weakly endothermic) (*T*_head_ = *T*_thorax_ > *T*_abdomen_; class *b* in Fig. 4). The decrease in classes *a* and *b* with *T*_e_ was compensated for by an increase in ectothermic classes *e* and *f* (*T*_head_ = *T*_thorax_ = *T*_abdomen_ and *T*_head_ < *T*_thorax_ = *T*_abdomen_) (Fig. 4, class *e*: 5–32.7%, class *f*: 5–12.5%). The relative number of endothermic bees with a difference of *T*_thorax_ − *T*_abdomen_ > 1 °C or > 2 °C increased sharply below start values of 30.9 °C and 32.3 °C, respectively (*xi* in Fig. 5). In ectothermic bees these start values were at 27.1 °C and 24.5 °C, respectively (Fig. 5). On the brood nest, mean *T*_comb_ was slightly lower next to endothermic bees than beside ectothermic ones only at *T*_e_ = 13.7 and 18.8 °C but not different at higher *T*_e_ (Fig. 3b, c; *P* < 0.005, *t* test).

### Bee density and evaporative cooling

**Fig. 6:**
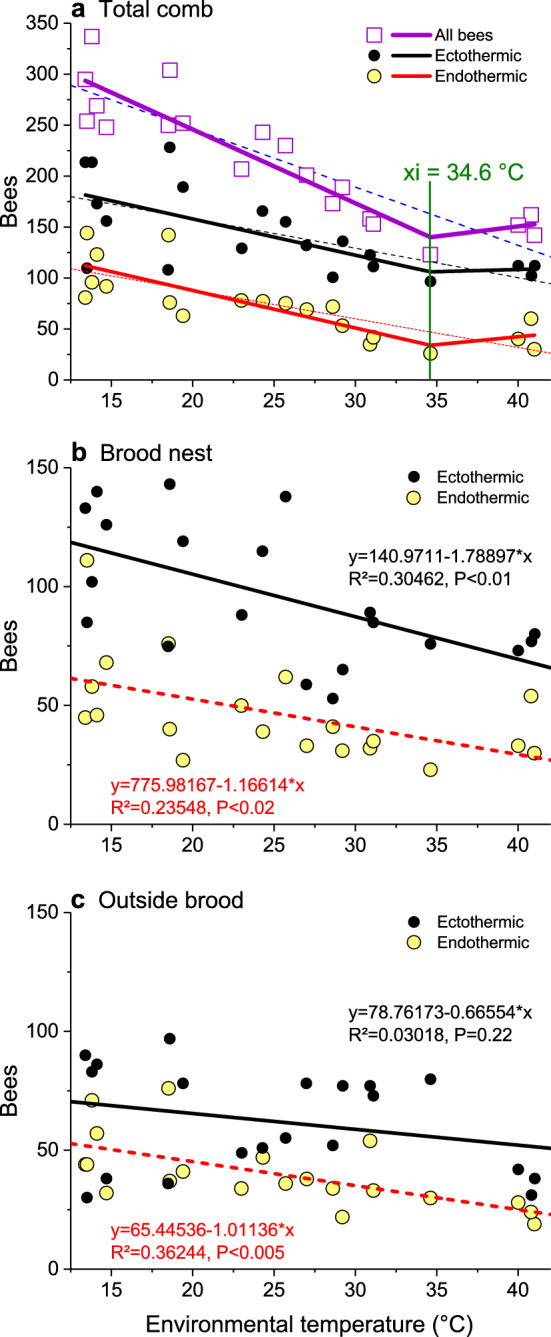
Change of number of bees classified as endothermic or ectothermic with environmental temperature (*T*_e_), on the total comb (**a**), on the brood nest (**b**) and outside it (**c**). *N* = 20 measurements; *R*^2^ adj. for df. **a** *P* values for piecewise and linear regressions, respectively: 0.8196 vs*.* 0.7797 (*P* << 0.0001 both) for all bees, 0.3446 vs*.* 0.39415 for ectothermic bees (*P* << 0.0001 vs*.* *P* < 0.005), and 0.6065 vs*.* 0.5289 in endothermic bees (*P* << 0.0001 both). Parameters for piecewise interpolation in (**a**) (*xi*: intersection point of regressions; for formula see legend of Fig. 5): *a*1 = 390.69272, *k*1 = -7.24397, *xi* = 34.6, *k*2 = 1.97065 for all bees; *a*1 = 229.11324, k1 = -3.55643, *xi* = 34.6, *k*2 = 0.42353 for ectothermic bees; *a*1 = 161.53877, *k*1 = -3.68596, *xi* = 34.6, *k*2 = 1.55034 for endothermic bees. Parameters for linear interpolation: *a* = 360.44429, *b* = -5.70865 for all bees; *a* = 216.06878, *b* = -2.89402 for ectothermic bees; *a* = 144.25341, *b* = -2.80977 for endothermic bees

**Fig. 7:**
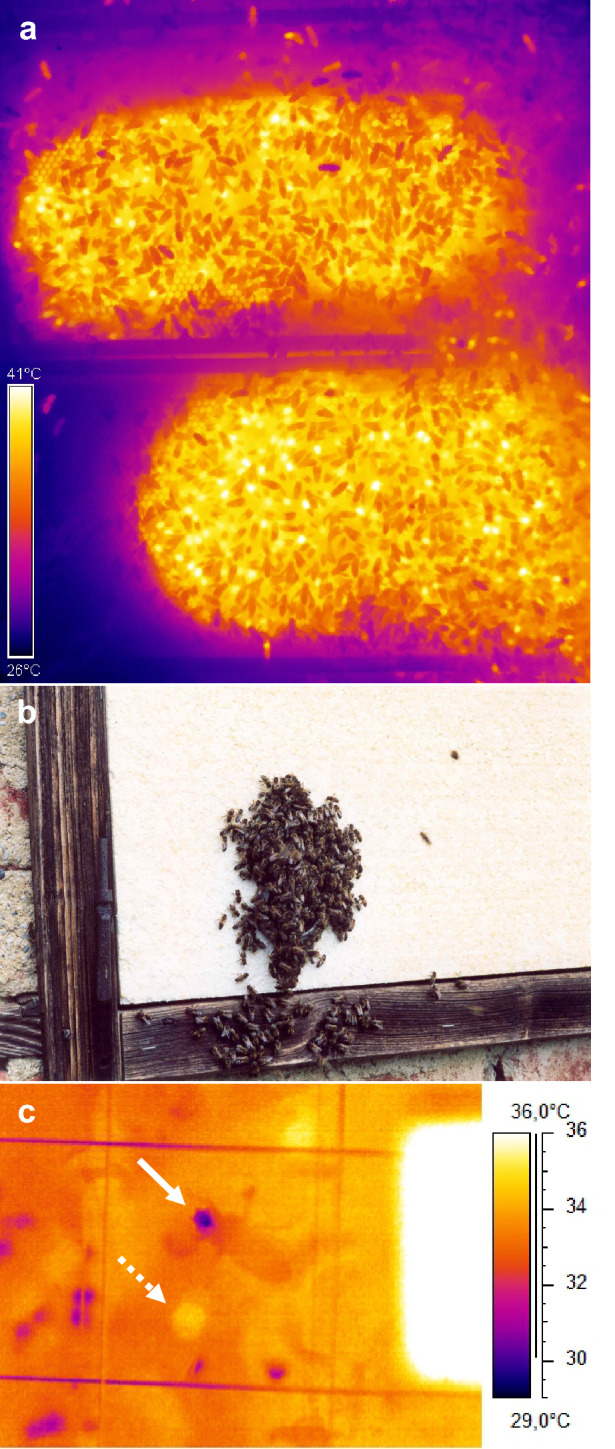
Bee behaviour during extreme cold (**a**) or heat stress (**b**, **c**). **a** Thermogram of a freezing summer colony in a 2-comb observation hive only covered by an infrared-transparent plastic film (*T*_e_ ~ 19 °C; montage of two thermograms). Note the cool bees on the upper comb forming an insulating layer, and the many endothermic bees visible as white spots. **b** Cluster outside the hive at *T*_e_ = 40 °C. **c** Thermogram of a bee with wet proboscis (arrow), an endothermic bee (dotted arrow), and cooling water droplets (left hand and bottom) at *T*_e_ = 40 °C. White area: reference radiator

**Fig. 8:**
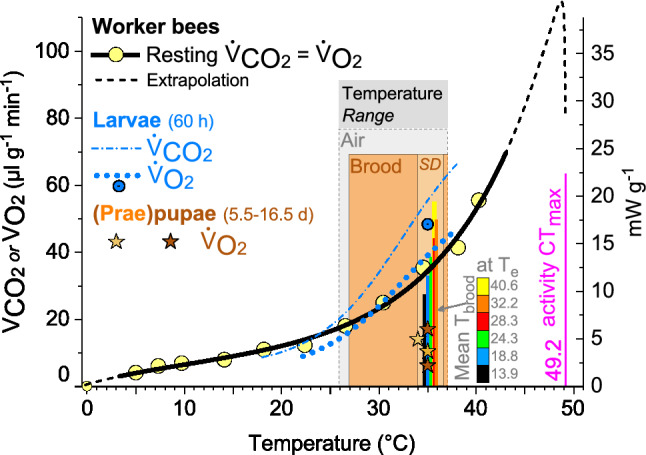
Honeybee worker resting metabolism (mean values, compiled from Kovac et al. 2007 and Kovac et al. 2014), and metabolism of larvae and (prae)pupae (from Petz et al. 2004; Melampy and Willis 1939) in dependence on ambient temperature (*T*_a_). Rectangles: range of air (grey) and brood surface temperature (orange); SD = temperature range from lowest to highest comb SD value in Fig. 3. Bars = mean *T*_brood_ at increasing *T*_e_, encoded by colours (see scale), and bar height for better differentiation. Critical thermal maximum (activity CT_max_) from Kovac et al. (2014). Worker resting VCO_2_ = *P*1 + (*P*2/(1 + *e*^*P*3−*P*4×T^)) + (*e*/(*T *− CT_max_)) − (*e*/(*P*5×ln(*T* − *P*7))); *T* = temperature (°C), *P*1 = 24.91394, *P*2 = 728,814.38656, *P*3 = 13.94319, *P*4 = 0.10574, *P*5 = 0.0433, CT_max_ = 49.2 °C, *P*7 = -12.4505. *R*^2^ = 0.98796, *P* < < 0.0001

**Fig. 9:**
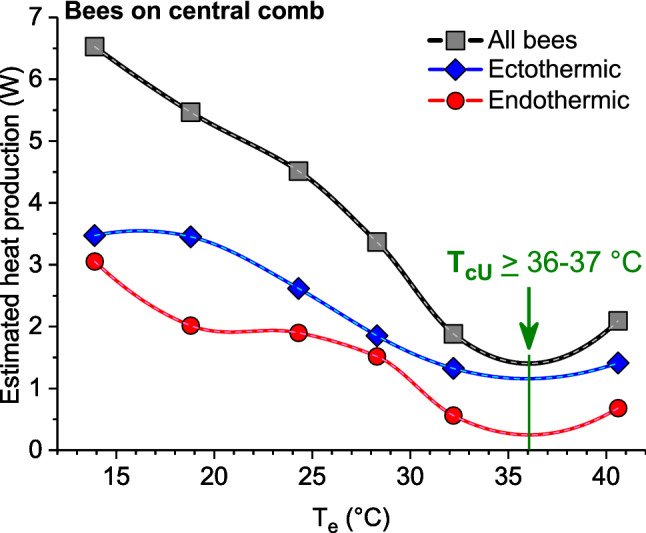
Estimated summed heat production of the bees on the central comb in dependence on environmental temperature (*T*_e_). Calculation of heat production on the basis of the measured mean body temperatures and numbers of ectothermic and endothermic bees present on the comb (compare Fig. 3); according to the resting CO_2_ curve from Fig. 8 for ectothermic bees, and the relation of energy turnover and body temperature of endothermic foragers (compiled from Stabentheiner and Kovac 2014)

A main mechanism in colony temperature homeostasis is the regulation of bee density. The total number and the number of both endothermic and ectothermic bees present on the comb increased significantly with decreasing *T*_e_ (Fig. 6a, b). Only in ectothermic bees outside the brood nest no change was observed (Fig. 6c). For the total comb, piecewise bipartite linear regressions described the relations more accurately than simple linear regressions (Fig. 6a). For the number of all bees on the comb, for example, *R*^2^ was 0.8196 and 0.7797, respectively. The piecewise interpolation indicated a minimum bee density at *xi* = 34.6 °C. In general, the number of bees classified as certainly endothermic (class *a* in Fig. 4) was much lower than of ectothermic bees (classes *b–i*; Figs. 4, 6). To reduce metabolic heat production further, at the highest environmental temperatures of about 40 °C, many bees left the colony to form a cluster outside the hive (Fig. 7b). Using the curve of honeybee resting metabolism (Fig. 8) and simultaneous measurement of forager respiration and body temperature (Stabentheiner and Kovac 2014), a model calculation of the approximate energetic investment of the bees present on the comb was performed (see Fig. 9). As was expected, adult bee metabolism increased approximately linearly with decreasing *T*_e_. Because of their larger number, ectothermic bees contributed more to heat production than the endothermic ones.

**Fig. 10:**
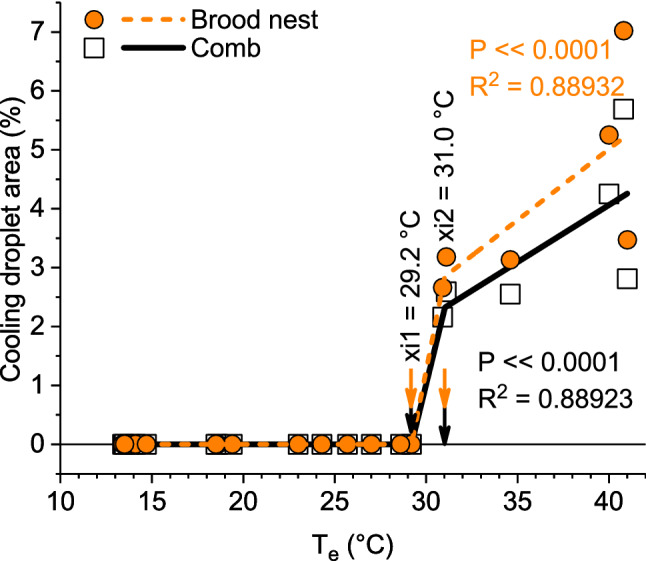
Percentage of brood nest or comb area covered by cooling spots in dependence on environmental temperature (*T*_e_). Interpolation with piecewise three-segment linear function: *yi*1 = *a*1 + *k*1 × *xi*1; *yi*2 = *yi*1 + *k*2 × (*xi*2 − *xi*1); if (*x* < *xi*1) *y* = *a*1 + *k*1 × *x*, else if (*x* < *xi*2) *y* = *yi*1 + *k*2 × (*x* − *xi*1), else *y* = *yi*2 + *k*3 × (*x* − *xi*2). *xi*1, *xi*2: intersection points of regressions. Parameters: *brood nest*: *a*1 = 1.46626×10^–9^, *k*1 = -7.31781×10^–11^, *xi*1 = 29.2, *xi*2 = 31.01881, *k*2 = 1.56471, *k*3 = 0.24133; *total comb*: *a*1 = 3.02688×10^–9^, *k*1 = -1.5103×10^–10^, *xi*1 = 29.2, *xi*2 = 31.02884, *k*2 = 1.27059, *k*3 = 0.19383. *N* = 20 measurements, *R*^2^ = adj. for df

Evaporative cooling of the brood started at environmental temperatures higher than 29–30 °C (Fig. 10, Table S1, dark spots in Figs. 1c, 7c). The maximum area covered by cool water droplets was 7% of the brood nest and 6% of the total central comb (Table S1). The local cooling effect in comparison to adjacent capped brood cells amounted from -0.6 to -4.6 °C, and tended to increase with increasing *T*_e_ (Table 1).

**Table 1:** Temperature of cooling water spots and nearby capped brood cells on the central comb during heat stress

*T*_e_ (°C)		Spot temperature (°C)	SD	Comb temperature (°C)	SD	*N*	Mean difference (°C)	Max difference (°C)	Min difference (°C)
30.9		34.3	0.8	35.9	0.3	51	-1.7	-3.3	-0.7
31.1		33.2	0.7	35.1	0.4	63	-2.0	-3.4	-0.6
34.6		33.9	0.7	35.6	0.4	110	-1.7	-3.6	-0.7
32.2	Mean	33.8	0.8	35.6	0.5	224	-1.8	-3.6	-0.6
40.0		32.6	0.8	34.6	0.4	94	-2.1	-4.6	-0.8
40.8		32.7	0.7	34.8	0.4	140	-2.2	-4.4	-0.6
41.0		34.6	0.7	36.6	0.3	107	-2.0	-3.4	-0.8
40.6	Mean	33.3	1.2	35.3	0.9	341	2.1	-4.6	-0.6

At environmental temperatures (*T*_e_) below 30 °C no spots were present (14 measurements at 4 ranges of *T*_e_, see Table S1 and Fig. 10)

## Discussion

A honeybee colony has to be regarded as a ‘superorganism’ where the cooperation of thousands of individuals makes the whole colony act in analogy to a multicellular organism (Moritz and Southwick 1992). This specific kind of cooperation is kept up not only during the breeding season but also in times of low yield of honey and pollen and low temperature (Moritz and Southwick 1992). Active cooperative thermoregulation of individuals is an essential requirement for honeybee survival, not just in temperate zones with cold winters and cool periods in the breeding season but also in hot climates (Abou-Shaara et al. 2017).

### Cooperative mechanisms of heat production

A distinct characteristic originating from warm climate is the regulation of a rather high core temperature (Koeniger 1978; Ritter 1982; Southwick 1985). Like in all poikilothermic insects, the respiratory metabolism, and thus speed of development, of the brood increase disproportionately with ambient temperature (Fig. 8) (Melampy and Willis 1939; Allen 1959; Petz et al. 2004). It appears that honeybees not only keep up their ‘tropical conditions’ in the brood nest even in temperate and cold regions. Our investigation demonstrates how they use a set of regulatory mechanisms to achieve not just a high but a constant speed of brood development by regulating mean capped brood temperature within 34.8–35.9 °C (*T*_e_ = 13–41 °C, Fig. 3), which compares favourably with earlier measurements in selected brood cells (Kraus et al. 1998) (34.8–35.8 °C; *T*_e_ = 18–32 °C). Bees strive to stabilise brood temperature in the first and air temperature in the second place (Koeniger 1978; Kronenberg and Heller 1982; Southwick 1985; Stabentheiner et al. 2010). This can be seen by the fact that the mean thorax temperature of endothermic bees, involved in active thermoregulation, was close to or higher than the comb temperature (Fig. 3b) while that of ectothermic bees was clearly lower (Fig. 3c). Outside the brood nest, thermoregulation is less accurate (Fig. 3) (Kraus et al. 1998; Stabentheiner et al. 2010). Active heat production, however, is not restricted to the brood nest. The thorax of endothermic bees was clearly warmer than the comb temperature in the peripheral comb areas (Fig. 3e). This heat is not wasted but reduces the heat flow out of the brood nest. It also guarantees a body temperature higher than 20 °C of peripheral (ectothermic) bees even in resting clusters below the combs (Figs. 2, 3). This is different to the winter season without brood where it is not so much the core temperature but the temperature of the surface bees which the core bees have to take care of, to prevent them of falling off the cluster (Stabentheiner et al. 2003a). If bees cool below the limiting temperature of about 10 °C their respiration (Lighton and Lovegrove 1990; Kovac et al. 2007) and muscular function fail (Esch 1988).

**Fig. 11:**
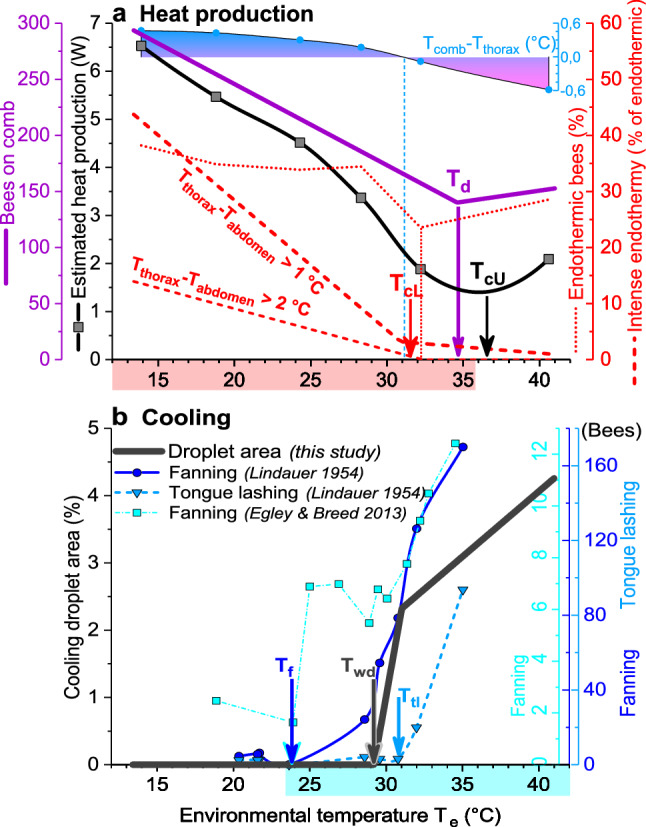
Synopsis of mechanisms of (**a**) heat production, and (**b**) hive cooling, and their setpoints (start values) in a honeybee colony. Ranges of regulation of heating and cooling overlap considerably (coloured bars on *x*-axes). *Setpoints*: **a T**_**d**_ = bee density (represented by bees on central comb), ***T***_**cL**_ and ***T***_**cU**_ = lower and upper critical temperatures, respectively; the relation between comb and bee thorax surface temperature changes close to ***T***_**cL**_ (compare Fig. 3). **b T**_**wd**_ = spreading of water droplets; ***T***_**f**_ = fanning and ***T***_**tl**_ = tongue-lashing (compiled from Lindauer 1954 and Egley and Breed 2013)

Honeybee colonies have to be seen as (quasi-)homeothermic organisms (Southwick 1982; Moritz and Southwick 1992), which try to regulate their core temperature at a constant level. In homeotherms, reaction to an increasing heat loss includes increasing insulation and, below a certain lower critical temperature (*T*_cL_), increasing metabolic heat production (Morgan 1998). The *T*_cL_ depends on the desired core temperature, on the mass and, thus, heat capacity, on the relation between heat exchanging surfaces and mass, on external and internal convection, and on the capability to regulate insulation. In broodless winter clusters the *T*_cL_ is as low as -5 to -10 °C because the bees increase insulation to a maximum at these temperatures (Southwick 1983, 1985; Heinrich 1993). For breeding summer colonies, our body temperature measurements disclose the unique possibility to define a lower critical temperature in analogy to homeothermic organisms like mammals and birds (Willmer et al. 2000). However, a colony cannot increase heat production at the flick of a switch because this decision is an individual one in the honeybee community (Moritz and Southwick 1992; Myerscough 1993; Watmough and Camazine 1995; Jones and Oldroyd 2007; Ocko and Mahadevan 2013). This is different to mammals and birds where the central nervous system regulates the heat production. Figure 11 provides a synopsis of the setpoints (start temperatures) of worker behaviour which help to define critical temperatures of the whole colony. A count of the percentage of endothermic bees (class *a* in Fig. 4) and of the percentage of them showing intense endothermy (Fig. 5) demonstrates a sharp increase below an environmental start temperature (*T*_e_) of 31–32 °C. This is the lower critical temperature (*T*_cL_) of a breeding honeybee colony. However, this does not mean that above this value there would be no active, endothermic heat production at all. Some bees still have a heated thorax, though endothermy is mostly weak (Figs. 4, 7c). We were not able to observe the behaviour of these bees in the few seconds of thermographic measurement, but foragers bringing in water for cooling at these temperatures (Figs. 10, 11) will enter the colony with a heated thorax, like all foragers (Schmaranzer 2000; Stabentheiner et al. 2007; Kovac et al. 2010, 2018). It has to be kept in mind, however, that the intensity of reactions necessary to compensate for changes in environmental temperature will change with colony strength (e.g. Southwick 1985) and properties of its nest insulation (Mitchell 2016) eviating from our colony setup.

In addition to the increase of endothermy, with decreasing *T*_e_ the bees move to the brood nest, and increase bee density there (Figs. 6, 11). This way, however, they not only increase insulation but also passive heat production, because bees must not just be seen as simple ‘technical insulators’ like mammalian fur or bird plumage. If they walk from the cooler periphery to the warm brood nest, this leads to a considerable increase of heat production because of the approximately exponential progression of their passive standard (resting) metabolism with ambient temperature (Fig. 8). Their increasing number reinforces this effect (Figs. 4, 6). A model calculation of the total heat production of the adults on the central comb, i.e. from the resting metabolic rate of ectothermic bees and the active metabolic rate of endothermic bees, shows that the combined effects of bee density and endothermy lead to a steep increase of heat production already below a *T*_e_ of about 34 °C (Fig. 9). In general, all age classes of bees have to be considered as ‘active isolators’ with respect to environmental changes. The very young ones preferably in terms of migration activity into or out of empty brood nest cells (if available), and the older ones by both migration and facultative endothermy (Stabentheiner et al. 2010). If present, also drones contribute to heat production and thermal homeostasis (Harrison 1987; Kovac et al. 2009). At even higher heat loss, as is the case in poorly insulated observation hives, not only many more intensely endothermic bees appear (Fig. 7a; Stabentheiner et al. 2010) but also the formation of an insulating bee layer becomes visible (Fig. 7a, upper comb).

### Prevention of heat stress

If a colony is in danger of overheating on warm days or if the sun is shining on it, the bees have to take cooling measures. We show that they are able to cool the comb temperature to below their body temperature both on and outside the brood nest even at the highest environmental temperatures (Figs. 3, 11a). The even lower air temperature in the space between the combs, always kept below 35.5 °C on average, guarantees a heat flow out of the comb. This is necessary, because the brood in the comb cells has no possibility to decrease its metabolism below the standard level. It is the adults who have to take care of this heat flow at high environmental temperatures, to lead off the metabolic heat of larvae and pupae to prevent overheating (Stabentheiner et al. 2010). One strategy to accomplish this is the reduction of bee density (number), which reaches a minimum at environmental temperatures of 34–36 °C (Figs. 6, 11a), beside reduction of endothermy (Figs. 4, 5). This way the heat produced in the bee spaces by ectothermic adults, which make up the majority of bees at high temperatures (classes *b–i* in Fig. 4), decreases with bee density. The reduction of their number to the amount necessary for brood care and cooling reduces the necessity of cooling measures. The resulting decrease of insulation to facilitate heat flow away from the brood, however, is in part counteracted by the increase of passive heat production (Fig. 8) by the increased body temperature of the bees (Figs. 2, 3). Bees were also reported to walk out of the brood nest towards the colony envelope, especially if the sun shining on a hive applies heavy heat stress (Starks and Gilley 1999; Johnson 2002; Starks et al. 2005). Such “heat shielding”, however, may not only delay the heat flow into the colony. It inevitably will increase passive (resting) heat production of those bees over time, because they are not just passive insulators. Their increasing body temperature will increase metabolic heat production automatically (Fig. 8). Therefore, bees even tend to leave the colony and form a cluster outside the hive entrance, known as “bee beard” by beekeepers, to minimise a further unwanted heat production inside (Fig. 7b).

A further important measure against overheating is evaporative cooling. Water gatherers collect water and spread droplets on the combs (Fig. 1, Fig. S2, Table S1) (Lindauer 1954; Kühnholz and Seeley 1997). In addition, hive bees tend to perform tongue-lashing during heat stress (Figs. 7c, 11b) (Lindauer 1954; Kühnholz and Seeley 1997). The main trigger for water collection is the demand in the hive, sensed by the water collectors via the unloading time to hive bees (Kühnholz and Seeley 1997). If the demand is high, water gatherers even start dancing to recruit helpers (Lindauer 1954). In our experiments, with the colony not exposed to the sun, cooling with water droplets started already at environmental temperatures higher than about 29–30 °C (Figs. 1, 10, 11, Fig. S2, Table S1), although heat production (Figs. 5, 9) and bee density (Fig. 6) were still at an increased level. This threshold is lower than the environmental temperature where the proportion of intensely endothermic bees reaches a minimum (31–32 °C, Fig. 5), and much lower than the temperature of minimum bee density (34–36 °C; Fig. 6). In hives exposed to the sun, evaporative cooling probably starts at even lower environmental temperatures.

Our findings raise the question of whether it is possible to define an upper critical temperature (*T*_cU_) of a honeybee colony comparable to homeotherms. In homeotherms, a common definition is as a specific ambient temperature at the upper end of the thermoneutral zone above which metabolic rate may again rise due to the direct effects of temperature on metabolic processes and the necessity of increased cooling efforts (Willmer et al. 2000). In mammals and birds, however, the definition of an upper critical temperature turned out to be ‘not amenable to the construction of an absolute definition’ (Morgan 1998). In horses, for example, it was determined as 20 °C, 25 °C or 30 °C, depending on whether it was estimated as the point where evaporative heat loss by sweating increased, metabolic rate increased or thermal insulation reached a minimum, respectively (Morgan 1998). In a honeybee colony, this would resemble the start of cooling with droplets (*T*_e_ ~ 29–30 °C; Fig. S2, Figs. 7c, 10), the increase of metabolic heat production because of increased cooling efforts (*T*_e_ ~ 36–37 °C; Fig. 9), or where bee density reached a minimum (*T*_e_ ~ 34–36 °C; Fig. 6). However, a metabolic estimation of the upper critical temperature as presented in Fig. 9 or by measurement of the total colony energy turnover will remain incomplete because it does not include the high energetic effort of water collectors (Schmaranzer 2000; Kovac et al. 2010, 2018), which start their cooling activity already at *T*_e_ > 29–30 °C (Fig. S2, Fig. 11) (Lindauer 1954). This comparison demonstrates that also in honeybee coloniesthere is no simple definition of an upper critical temperature, because regulatory mechanisms have different setpoints.

Another mechanism to prevent overheating is the regulation of fanning activity (Lindauer 1954; Southwick 1985; Sudarsan et al. 2012; Cook and Breed 2013; Egley and Breed 2013; Cook et al. 2016). Fanning is one of the first behaviours bees start under heat stress (Lindauer 1954; Johnson 2002), comparable to bumblebees (Weidenmüller et al. 2002, Weidenmüller 2004). While fanning activity is always present for rhythmic gas exchange and concentration of nectar at moderate thermal conditions (Seeley 1974; Southwick and Moritz 1987), the bees increase it under heat stress already at temperatures higher than 24–25 °C (Lindauer 1954; Kronenberg and Heller 1982; Egley and Breed 2013) (see Fig. 11), which is well below the start of cooling with water droplets found in our study (> 29–30 °C; Table 1, Figs. 10, 11). It improves in-hive heat transport by convection and, in addition, helps to remove water-saturated air from the combs. A recent investigation showed that bees try to establish a directed stream of airflow into and out of the colony, which guarantees optimal air circulation (Peters et al. 2019). It has to be kept in mind that fanning bees are always endothermic because their flight muscles are active but nevertheless contribute to colony cooling (our unpublished observation). Tongue lashing as an additional measure of cooling (Fig. 7c) starts only at temperatures higher than 31 °C (Lindauer 1954; see Fig. 11). These cooling activities start already at moderate temperatures where heat production and bee density are still at an increased level (Fig. 11).

### Benefit of interlaced regulatory mechanisms

An important result of the present study is that mechanisms of colony heating and cooling overlap in a broad environmental temperature range (Fig. 11). Several strongly interlaced regulatory mechanisms and passive effects prevent cooling or overheating of the brood. Setpoints (starting points) of different regulatory behaviours vary considerably. From control theory, with the knowledge in mind that counteraction to environmental changes takes place at the individual level, the variation of starting points of behaviours makes sense. The many sensory and regulatory units (bees) are distributed all over the colony. Changes of heat flow due to environmental changes will reach them with differing delay. The air temperature between the combs is not constant but may fluctuate considerably over time (see supplementary Video S1). Individuals need time to sense and to integrate temperature changes, and to react properly (Seeley 1974; Johnson 2002, 2008), and bees probably differ in their thresholds of task onset (Myerscough 1993; Watmough and Camazine 1995; Jones and Oldroyd 2007). Starting cooling behaviours already at temperatures where heating is not yet at a minimum (and vice versa) will stabilise in-hive climate. However, active counter-cooling at temperatures where heating mechanisms are still active is energetically costly. Honeybee colonies can afford this, because their energy-intensive style of living (e.g. Stabentheiner and Kovac 2014, 2016) rests on their access to large resources of energy from nectar and honeydew, and on their storage economy, which helps them to overcome times of poor energetic income (yield) with stockpiled reserves (Seeley 1995).

## Conclusion

Honeybee colonies make good use of a set of behavioural and physiological regulatory mechanisms and passive effects for brood temperature homeostasis. The different regulatory mechanisms are interlaced and change together in quite a broad range of environmental conditions. Our comprehensive analysis promotes a better understanding of the interaction and importance of the various regulatory mechanisms.

## Supplementary Information

Below is the link to the electronic supplementary material.

Supplementary file1 (PDF 1,932 KB)

Supplementary file2 (MP4 24,232 KB)
