# Supplementary material for: Coping with the cold and fighting the heat: thermal homeostasis of a superorganism, the honeybee colony
Source: J Comp Physiol A Neuroethol Sens Neural Behav Physiol. 2021 Feb 17;207(3):337–51. doi: 10.1007/s00359-021-01464-8 (PMC8079341; doi:10.1007/s00359-021-01464-8)
Supplement: Supplementary file 1 — Supplementary file1 (PDF 1,932 KB) [file 359_2021_1464_MOESM1_ESM.pdf]

Supplementary material to

Coping with the cold and fighting the heat: thermal homeostasis of a superorganism, the honeybee colony

Anton Stabentheiner, Helmut Kovac, Monika Mandl, Helmut Käfer

Institute of Biology, University of Graz, Austria

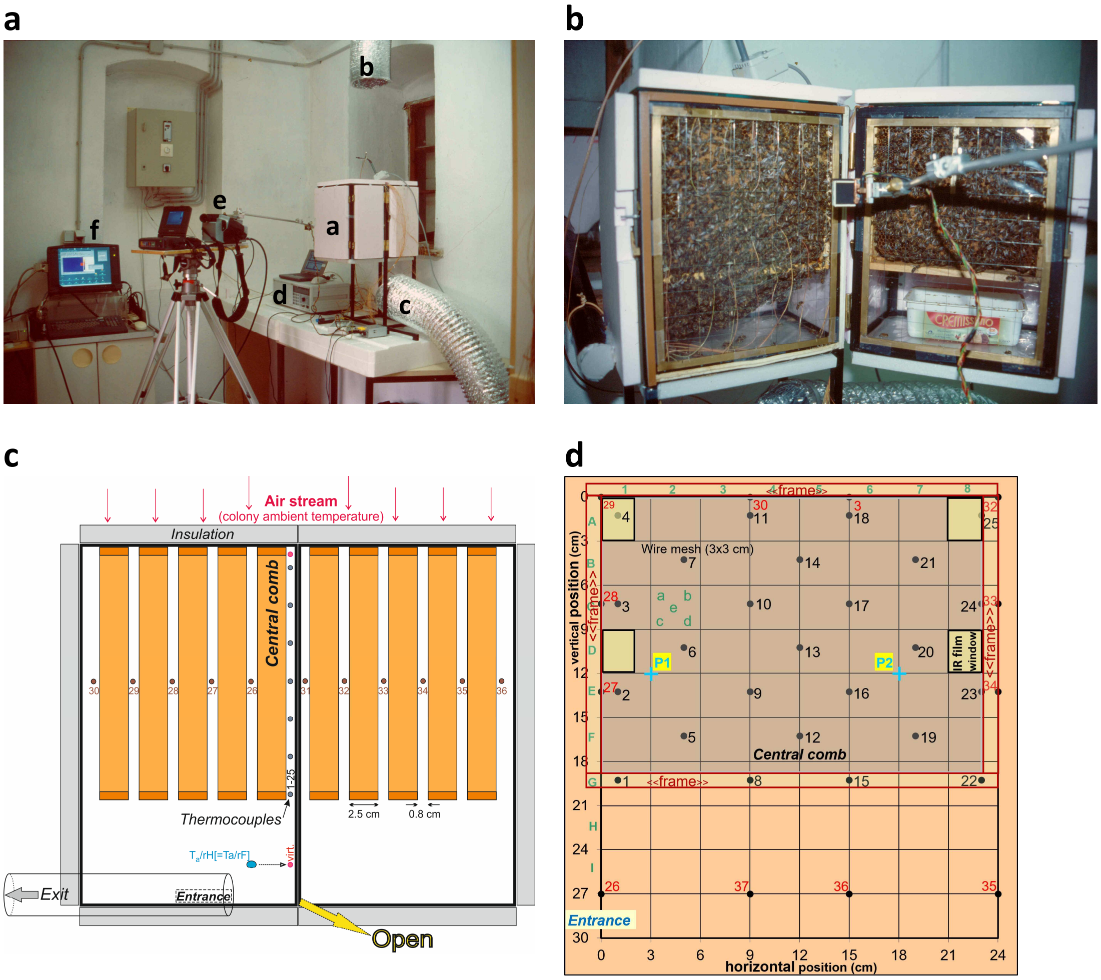

Fig. S1 Experimental setup. The cold or heat stress was varied by placing the hive in an air stream at temperatures of 13.5, 19, 24, 28.5, 32.2 or 40 °C (T_e = environmental temperature). **a** Insulated honeybee hive (40x31x36 cm) (a), air inflow (b) and withdrawal (c), data logger with laptop (d), IR camera (e), PC for IR recording (f). **b** Experimental hive opened between central combs, with IR-transmitting films covering the combs; black square: reference radiator. **c** Scheme of hive with combs, thermocouple alignment and T/rH sensor in air space below combs. **d** Thermocouple assembly (black numbers) on central comb (grey area); red numbers: virtual temperature measurement points (nearest neighbour thermocouples on comb or T/rH sensor below combs) for triangular interpolation of air temperature at bee position (T_a); light yellow rectangles: communication windows between hive-halves cut out from the covering IR-transmitting films. P1, P2: measurement points for size calibration of thermograms.

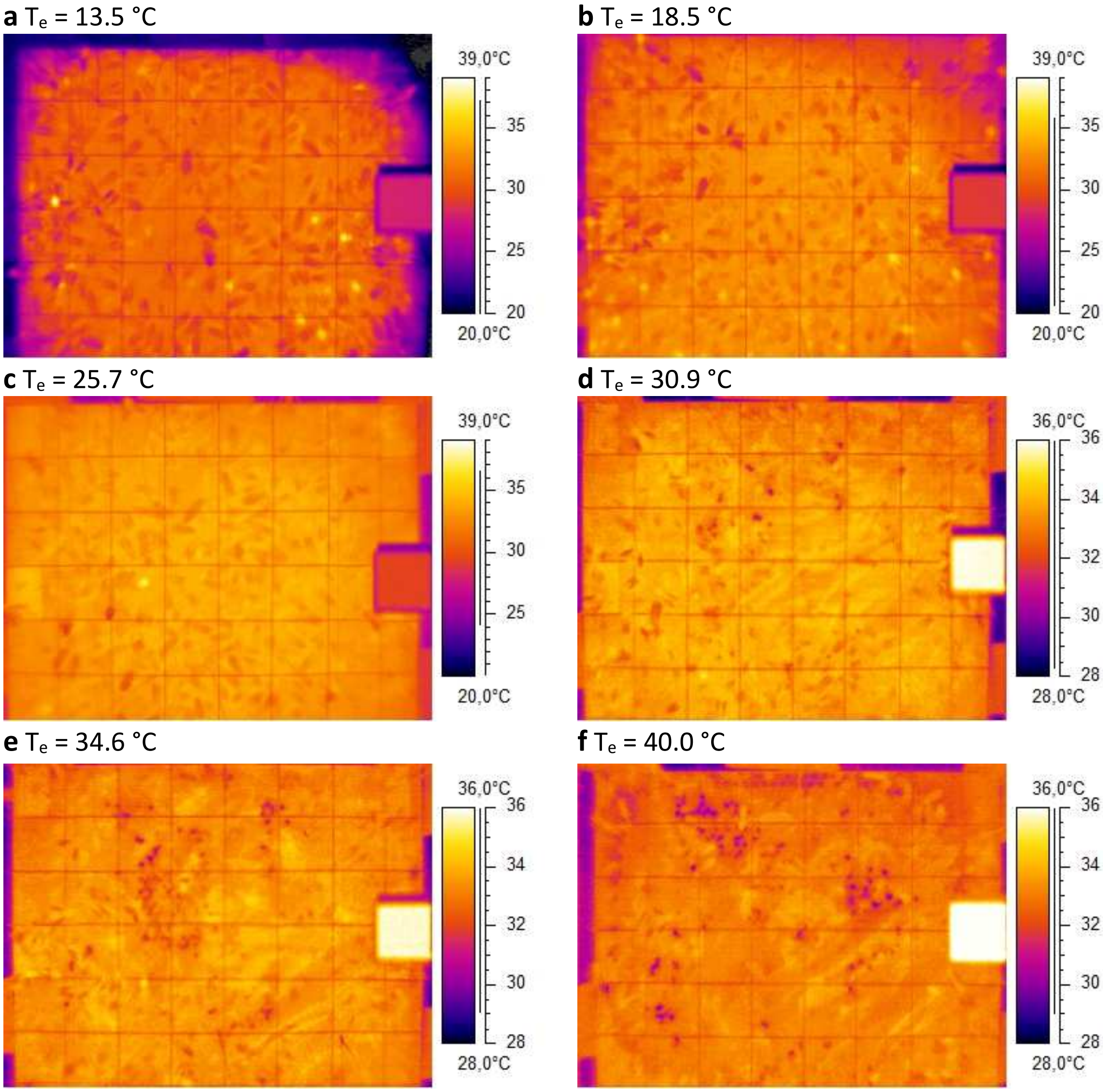

Fig. S2 Sample thermograms of the central comb of an isolated standard colony at various environmental temperatures (T_e). Note intensely endothermic bees (yellow spots) in (**a**), and dark spots in (**d**), (**e**) and (**f**) where bees had spread water for cooling of the comb. Lines: wire mesh for position determination; right-hand squares: reference radiator for IR-camera calibration.

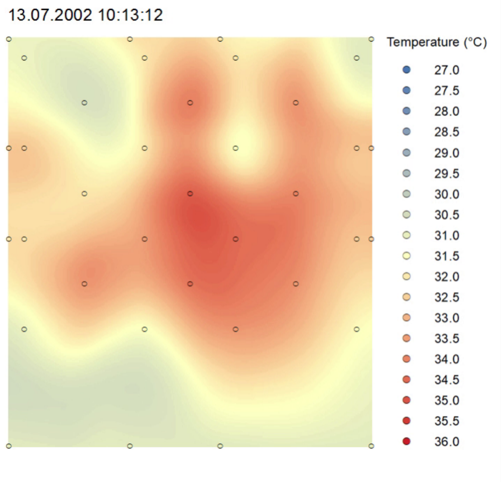

Video S1 Temporal fluctuations of air temperature (T_a) on the central comb of an undisturbed 10-comb honeybee colony during a 95 min period, at an environmental temperature (T_e) of 23.9 °C. See file “**Video-S1_Stabentheiner et al. 2020.mp4**”.

Table S1 Number of cooling water spots on central comb during heat stress (see Figs 1, 7, 10, S2). At environmental temperatures (T_e) below 30 °C no spots were present (14 measurements at 4 ranges of T_e).

**T_e** (°C)	**Time of day** (hh:min)	**Cool spots** (number)	**Area of brood nest** (%)	**Mean** (%)	SD	**Area of comb** (%)	**Mean** (%)	SD	N
<30		0	0	0		0	0		14
34.6	10:00	114	3.13			2.55			
30.9	12:00	51	2.66			2.16			
31.1	14:00	63	3.18	2.99	0.287	2.59	2.43	0.238	3
41.0	10:00	140	3.47			2.81			
40.8	12:00	139	7.02			5.69			
40.0	14:00	94	5.25	5.25	1.775	4.25	4.25	1.44	3

Table S2, a Statistics for comparison of body temperatures, comb surface temperatures and air temperatures shown in Fig. 3. T_e = environmental temperature, T_a = air temperature next to bees. All temperatures in °C.

t-Test	a) BROOD NEST										
**Compared values**			**Values:**								
**Value1**	**Value2**	At	**Mean1**	SD1	N1	**Mean2**	SD2	N2	***t* value**	***df***	**P < ......**
ALL Bees Brood		**T_e (°C)**									
T_{thorax}	T_{comb}	13.9	**34.3**	1.27	914	**34.8**	0.69	914	**9.752209712**	1826	0.0001
		18.8	**34.6**	0.98	480	**35.1**	0.54	480	**8.347241111**	958	0.0001
		24.3	**35.1**	0.87	492	**35.4**	0.74	492	**5.906339701**	982	0.0001
		28.3	**35.6**	0.85	282	**35.8**	0.65	282	**2.723126283**	562	0.01
		32.2	**36.0**	0.54	340	**35.9**	0.51	340	**2.144514046**	678	0.05
		40.6	**36.3**	0.88	347	**35.7**	0.98	347	**8.311908983**	692	0.0001
T_{thorax}	T_a	13.9	**34.3**	1.27	914	**31.6**	1.84	914	**36.50293258**	1826	0.0001
		18.8	**34.6**	0.98	480	**32.7**	0.99	480	**31.01959838**	958	0.0001
		24.3	**35.1**	0.87	492	**32.8**	1.05	492	**37.96718821**	982	0.0001
		28.3	**35.6**	0.85	282	**33.5**	1.02	282	**26.51439479**	562	0.0001
		32.2	**36.0**	0.54	340	**34.6**	1.03	340	**21.1924827**	678	0.0001
		40.6	**36.3**	0.88	347	**34.9**	1.56	347	**14.9435638**	692	0.0001
T_{comb}	T_a	13.9	**34.8**	0.69	914	**31.6**	1.84	914	**48.5895399**	1826	0.0001
		18.8	**35.1**	0.54	480	**32.7**	0.99	480	**46.68753028**	958	0.0001
		24.3	**35.4**	0.74	492	**32.8**	1.05	492	**45.45889516**	982	0.0001
		28.3	**35.8**	0.65	282	**33.5**	1.02	282	**31.46015609**	562	0.0001
		32.2	**35.9**	0.51	340	**34.6**	1.03	340	**20.07892717**	678	0.0001
		40.6	**35.7**	0.98	347	**34.9**	1.56	347	**8.579374606**	692	0.0001
ENDOTHERMIC bees Brood		**T_e (°C)**									
T_{thorax}	T_{comb}	13.9	**34.6**	1.69	328	**34.7**	0.78	328	**0.624315822**	654	n.s.
		18.8	**35.0**	1.29	143	**34.9**	0.63	143	**0.529784537**	284	n.s.
		24.3	**35.4**	1.04	151	**35.4**	0.74	151	**0.204005023**	300	n.s.
		28.3	**35.9**	0.96	105	**35.9**	0.69	105	**0.460170504**	208	n.s.
		32.2	**36.2**	0.56	90	**35.9**	0.49	90	**3.183657166**	178	0.002
		40.6	**36.4**	0.84	117	**35.6**	0.96	117	**6.482009812**	232	0.0001
T_{thorax}	T_a	13.9	**34.6**	1.69	328	**31.3**	1.91	328	**23.63947636**	654	0.0001
		18.8	**35.0**	1.29	143	**32.5**	0.98	143	**18.23409319**	284	0.0001
		24.3	**35.4**	1.04	151	**32.8**	1.09	151	**21.53605137**	300	0.0001
		28.3	**35.9**	0.96	105	**33.5**	1.04	105	**17.51726385**	208	0.0001
		32.2	**36.2**	0.56	90	**34.5**	1.08	90	**12.92697283**	178	0.0001
		40.6	**36.4**	0.84	117	**34.8**	1.39	117	**10.3809997**	232	0.0001
T_{comb}	T_a	13.9	**34.7**	0.78	328	**31.3**	1.91	328	**29.81218135**	654	0.0001
		18.8	**34.9**	0.63	143	**32.5**	0.98	143	**24.66972309**	284	0.0001
		24.3	**35.4**	0.74	151	**32.8**	1.09	151	**24.35243806**	300	0.0001
		28.3	**35.9**	0.69	105	**33.5**	1.04	105	**19.40458032**	208	0.0001
		32.2	**35.9**	0.49	90	**34.5**	1.08	90	**11.27218893**	178	0.0001
		40.6	**35.6**	0.96	117	**34.8**	1.39	117	**5.081376934**	232	0.0001
ECTOTHERMIC bees Brood		**T_e (°C)**									
T_{thorax}	T_{comb}	13.9	**34.2**	0.91	586	**34.9**	0.64	586	**15.05034088**	1170	0.0001
		18.8	**34.5**	0.77	337	**35.1**	0.49	337	**12.80492291**	672	0.0001
		24.3	**35.0**	0.75	341	**35.5**	0.74	341	**7.859547677**	680	0.0001
		28.3	**35.5**	0.73	177	**35.8**	0.62	177	**4.282981093**	352	0.0001
		32.2	**35.9**	0.51	248	**35.9**	0.51	248	**0.541487395**	494	n.s.
		40.6	**36.3**	0.89	230	**35.8**	0.98	230	**5.643594182**	458	0.0001
T_{thorax}	T_a	13.9	**34.2**	0.91	586	**31.8**	1.78	586	**28.44460412**	1170	0.0001
		18.8	**34.5**	0.77	337	**32.7**	0.99	337	**25.82041327**	672	0.0001
		24.3	**35.0**	0.75	341	**32.8**	1.03	341	**31.79092171**	680	0.0001
		28.3	**35.5**	0.73	177	**33.6**	1.02	177	**20.31620747**	352	0.0001
		32.2	**35.9**	0.51	248	**34.7**	0.99	248	**17.02588339**	494	0.0001
		40.6	**36.3**	0.89	230	**34.9**	1.64	230	**11.12765736**	458	0.0001
T_{comb}	T_a	13.9	**34.9**	0.64	586	**31.8**	1.78	586	**38.93163639**	1170	0.0001
		18.8	**35.1**	0.49	337	**32.7**	0.99	337	**39.90750299**	672	0.0001
		24.3	**35.5**	0.74	341	**32.8**	1.03	341	**38.38886206**	680	0.0001
		28.3	**35.8**	0.62	177	**33.6**	1.02	177	**24.73157062**	352	0.0001
		32.2	**35.9**	0.51	248	**34.7**	0.99	248	**16.63968614**	494	0.0001
		40.6	**35.8**	0.98	230	**34.9**	1.64	230	**6.935857769**	458	0.0001

Table S2, b Statistics for comparison of body temperatures, comb surface temperatures and air temperatures shown in Fig. 3. T_e = environmental temperature, T_a = air temperature next to bees. All temperatures in °C.

**t-Test**	**b) OUTSIDE BROOD NEST**										
**Compared values**			**Values:**								
**Value1**	**Value2**	At	**Mean1**	SD1	N1	**Mean2**	SD2	N2	***t* value**	***df***	**P < ......**
ALL Bees Outside		T_e (°C)									
T_{thorax}	T_{comb}	13.9	**31.9**	2.13	575	**32.3**	2.43	575	**3.436620268**	1148	0.001
		18.8	**33.4**	2.07	365	**33.4**	1.68	365	**0.007528171**	728	n.s.
		24.3	**34.3**	1.23	272	**34.2**	1.17	272	**0.584351438**	542	n.s.
		28.3	**35.1**	0.89	301	**35.3**	0.80	301	**2.822962322**	600	0.01
		32.2	**35.7**	0.55	134	**35.3**	0.66	134	**5.761644644**	266	0.0001
		40.6	**36.3**	0.78	182	**35.7**	0.86	182	**7.010525824**	362	0.0001
T_{thorax}	T_a	13.9	**31.9**	2.13	575	**29.5**	1.77	575	**20.58251123**	1148	0.0001
		18.8	**33.4**	2.07	365	**30.8**	1.33	365	**19.79846163**	728	0.0001
		24.3	**34.3**	1.23	272	**32.1**	0.98	272	**23.13178319**	542	0.0001
		28.3	**35.1**	0.89	301	**33.2**	1.04	301	**24.33349751**	600	0.0001
		32.2	**35.7**	0.55	134	**34.2**	0.89	134	**16.27520505**	266	0.0001
		40.6	**36.3**	0.78	182	**35.0**	1.55	182	**10.17688635**	362	0.0001
T_{comb}	T_a	13.9	**32.3**	2.43	575	**29.5**	1.77	575	**22.65244476**	1148	0.0001
		18.8	**33.4**	1.68	365	**30.8**	1.33	365	**22.67634607**	728	0.0001
		24.3	**34.2**	1.17	272	**32.1**	0.98	272	**23.16353694**	542	0.0001
		28.3	**35.3**	0.80	301	**33.2**	1.04	301	**27.95240224**	600	0.0001
		32.2	**35.3**	0.66	134	**34.2**	0.89	134	**10.96397552**	266	0.0001
		40.6	**35.7**	0.86	182	**35.0**	1.55	182	**5.377398997**	362	0.0001
ENDOTHERMIC bees Outside		T_e (°C)									
T_{thorax}	T_{comb}	13.9	**32.2**	2.25	248	**32.1**	2.67	248	**0.792779687**	494	n.s.
		18.8	**34.1**	2.40	154	**33.4**	1.86	154	**2.873111257**	306	0.01
		24.3	**34.9**	1.21	117	**34.3**	1.16	117	**3.877454832**	232	0.001
		28.3	**35.3**	0.90	94	**35.3**	0.80	94	**0.134775895**	186	n.s.
		32.2	**36.0**	0.49	41	**35.4**	0.62	41	**5.306811436**	80	0.0001
		40.6	**36.5**	0.80	71	**35.7**	0.76	71	**5.61442732**	140	0.0001
T_{thorax}	T_a	13.9	**32.2**	2.25	248	**29.4**	1.87	248	**15.16006631**	494	0.0001
		18.8	**34.1**	2.40	154	**30.8**	1.39	154	**14.45676124**	306	0.0001
		24.3	**34.9**	1.21	117	**32.2**	0.99	117	**18.84594902**	232	0.0001
		28.3	**35.3**	0.90	94	**33.2**	1.05	94	**15.18711652**	186	0.0001
		32.2	**36.0**	0.49	41	**34.3**	0.86	41	**11.04201395**	80	0.0001
		40.6	**36.5**	0.80	71	**34.9**	1.61	71	**7.571446333**	140	0.0001
T_{comb}	T_a	13.9	**32.1**	2.67	248	**29.4**	1.87	248	**12.7577793**	494	0.0001
		18.8	**33.4**	1.86	154	**30.8**	1.39	154	**13.51570046**	306	0.0001
		24.3	**34.3**	1.16	117	**32.2**	0.99	117	**15.05014067**	232	0.0001
		28.3	**35.3**	0.80	94	**33.2**	1.05	94	**15.79038394**	186	0.0001
		32.2	**35.4**	0.62	41	**34.3**	0.86	41	**6.366185086**	80	0.0001
		40.6	**35.7**	0.76	71	**34.9**	1.61	71	**4.176992722**	140	0.0001
ECTOTHERMIC bees Outside		T_e (°C)									
T_{thorax}	T_{comb}	13.9	**31.6**	2.00	327	**32.5**	2.23	327	**5.734087946**	652	0.0001
		18.8	**32.8**	1.60	211	**33.3**	1.55	211	**3.367099138**	420	0.001
		24.3	**33.9**	1.04	155	**34.2**	1.17	155	**2.76445414**	308	0.01
		28.3	**35.0**	0.86	207	**35.3**	0.80	207	**3.544936249**	412	0.0001
		32.2	**35.6**	0.53	93	**35.2**	0.53	93	**4.201550602**	184	0.0001
		40.6	**36.2**	0.75	111	**35.7**	0.92	111	**4.574302772**	220	0.0001
T_{thorax}	T_a	13.9	**31.6**	2.00	327	**29.5**	1.70	327	**14.15340267**	652	0.0001
		18.8	**32.8**	1.60	211	**30.8**	1.30	211	**14.47512545**	420	0.0001
		24.3	**33.9**	1.04	155	**32.0**	0.98	155	**15.78629711**	308	0.0001
		28.3	**35.0**	0.86	207	**33.2**	1.03	207	**19.23968531**	412	0.0001
		32.2	**35.6**	0.53	93	**34.2**	0.53	93	**17.67768442**	184	0.0001
		40.6	**36.2**	0.75	111	**35.1**	1.50	111	**6.945905049**	220	0.0001
T_{comb}	T_a	13.9	**32.5**	2.23	327	**29.5**	1.70	327	**19.3836288**	652	0.0001
		18.8	**33.3**	1.55	211	**30.8**	1.30	211	**18.47003842**	420	0.0001
		24.3	**34.2**	1.17	155	**32.0**	0.98	155	**17.58371697**	308	0.0001
		28.3	**35.3**	0.80	207	**33.2**	1.03	207	**23.01236117**	412	0.0001
		32.2	**35.2**	0.53	93	**34.2**	0.53	93	**13.47613382**	184	0.0001
		40.6	**35.7**	0.92	111	**35.1**	1.50	111	**3.540233954**	220	0.001
